# Spatial Distribution of Air Pollution, Hotspots and Sources in an Urban-Industrial Area in the Lisbon Metropolitan Area, Portugal—A Biomonitoring Approach

**DOI:** 10.3390/ijerph19031364

**Published:** 2022-01-26

**Authors:** Leonor Abecasis, Carla A. Gamelas, Ana Rita Justino, Isabel Dionísio, Nuno Canha, Zsofia Kertesz, Susana Marta Almeida

**Affiliations:** 1 Centro de Ciências e Tecnologias Nucleares, Instituto Superior Técnico, Universidade de Lisboa, Estrada Nacional 10, 2695-066 Bobadela, Portugal; maria.leonor.rente.abecasis@tecnico.ulisboa.pt (L.A.); carla.gamelas@ctn.tecnico.ulisboa.pt (C.A.G.); ana.justino@tecnico.ulisboa.pt (A.R.J.); dionisio@ctn.tecnico.ulisboa.pt (I.D.); smarta@ctn.tecnico.ulisboa.pt (S.M.A.); 2 ESTSetúbal/IPS and CINEA, IPS Campus, Polytechnic Institute of Setúbal, 2914-508 Setúbal, Portugal; 3 CESAM—Centre for Environmental and Marine Studies, Department of Environment and Planning, University of Aveiro, 3810-193 Aveiro, Portugal; 4 Laboratory for Heritage Science, Institute for Nuclear Research, H-4026 Debrecen, Hungary; zsofi@atomki.hu

**Keywords:** air pollution, biomonitoring, transplanted lichens, spatial analysis, urban-industrial area, steelworks, source apportionment

## Abstract

This study aimed to understand the influence of industries (including steelworks, lime factories, and industry of metal waste management and treatment) on the air quality of the urban-industrial area of Seixal (Portugal), where the local population has often expressed concerns regarding the air quality. The adopted strategy was based on biomonitoring of air pollution using transplanted lichens distributed over a grid to cover the study area. Moreover, the study was conducted during the first period of national lockdown due to COVID-19, whereas local industries kept their normal working schedule. Using a set of different statistical analysis approaches (such as enrichment and contamination factors, Spearman correlations, and evaluation of spatial patterns) to the chemical content of the exposed transplanted lichens, it was possible to assess hotspots of air pollution and to identify five sources affecting the local air quality: (i) a soil source of natural origin (based on Al, Si, and Ti), (ii) a soil source of natural and anthropogenic origins (based on Fe and Mg), (iii) a source from the local industrial activity, namely steelworks (based on Co, Cr, Mn, Pb, and Zn); (iv) a source from the road traffic (based on Cr, Cu, and Zn), and (v) a source of biomass burning (based on Br and K). The impact of the industries located in the study area on the local air quality was identified (namely, the steelworks), confirming the concerns of the local population. This valuable information is essential to improve future planning and optimize the assessment of particulate matter levels by reference methods, which will allow a quantitative analysis of the issue, based on national and European legislation, and to define the quantitative contribution of pollution sources and to design target mitigation measures to improve local air quality.

## 1. Introduction

Industrial emissions have an important contribution to particulate matter (PM) in urban-industrial areas [1,2]. To solve environmental problems in this type of areas it is crucial to understand and identify prevailing emission sources to promote targeted and successful mitigation measures.

It is known that particulate matter may have adverse health effects, since PM may contain potentially toxic elements (PTEs) [3,4,5]. Thus, the determination of the chemical composition of PM aims not only to determine the sources of the PM sampled at receptor sites but also to identify its potential health hazards.

In the last years, the episodic deposition of airborne particulates onto homes and properties was a common complaint from the residents of Aldeia de Paio Pires-Seixal (municipality of Seixal of the Lisbon metropolitan area), an urban-industrial area in Portugal [6]. To answer the concerns raised by the population, the local Council of Seixal promoted a set of actions, such as the assessment of the chemical composition of the settled dust to determine its sources and potential health hazards [7]. In this previous study, the settled dust was collected in January 2019, characterized by micro-PIXE, and the influence of the steel industries was identified due to the content of Fe, Cr, and Mn, along with a minor traffic influence.

PM emissions from steelworks are a complex mixture of stationary and diffuse emissions, associated with the process and with operations such as stocking and transportation of raw materials and slags [1,8,9]. Atmospheric PM from iron and steel industries have high concentrations of As, Cd, Cr, Cu, Fe, Mn, Ni, Se, V, and Zn [10,11,12]. Moreover, steelworks are often close to other industries and traffic zones, making it difficult to distinguish between the contributions of the processes. 

Traditionally, air pollution studies are performed through instrumental techniques that are limited to a small number of sampling stations [13]. Biomonitoring offers several advantages over standard sampling methods since biomonitors can be used in vast areas with many monitoring points while requiring little maintenance and are low cost [14]. Biomonitors can be native organisms in the ecosystem or they can be collected in an unpolluted site and transplanted to the area of interest [15,16].

Lichens have the ability to accumulate elements, in correlation with atmospheric levels. In fact, since lichens have no roots, they are dependent on the atmosphere for the uptake of water and mineral substances [17]. Lichens are extensively used in biomonitoring studies [13,18,19,20,21,22] and for spatial mapping of air contaminants [14,23,24], allowing the identification of pollution hotspots.

Continuing the set of actions promoted by the local Council of Seixal to understand the population exposure and the associated health risks of the settled dust events, the present study was conducted for the Council of Seixal and it aimed to assess the spatial distribution of air pollution, to identify its hotspots and its sources, by the use of the biomonitoring technique of air pollution using transplanted lichens in the Aldeia de Paio Pires-Seixal. The elemental characterization of the transplanted lichens was performed to assess the potential pollution sources, through the analysis of their chemical tracers. The Micro-X-ray Fluorescence technique was applied since it has been previously used to quantify metals in lichens and has several advantages: it is nondestructive, presents no need for sample digestion, and accuracy and reproducibility are at least equal to spectroscopic methods [25]. 

## 2. Materials and Methods

### 2.1. Study Area

**Figure 1:**
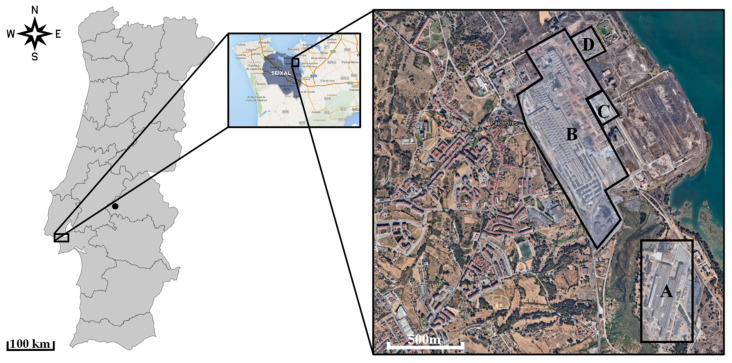
Location of the study area: (**left**) Framework of the study area (black rectangle) and the clean area (black dot, Montargil) in Portugal mainland; (**center**) Seixal municipality location; (**right**) location of industries A, B, C and D within the study area (geographical data obtained from Google Earth Pro [30]).

This study was carried out in the parish of União das Freguesias do Seixal, Arrentela e Aldeia de Paio Pires (UFSAAPP), in the municipality of Seixal (Portugal), which is located in the peninsula of Setúbal and it is a part of the Lisbon Metropolitan Area (Portugal), next to the Nature Reserve of the Tagus Estuary (Figure 1). The municipality of Seixal is one of the most densely populated municipalities in Portugal, with 167,294 inhabitants in 95.5 Km^2^ [26] (PORDATA, 2021).

Besides including a significant network of highways and national roads (A2-IP7, A33, and EN10), the study area (UFSAAPP) comprises small and medium-sized industries and an industrial park where the following facilities are located (as shown in Figure 1): industry A–that is a steelwork that manufactures galvanized sheet metal (with Cr passivation) and cold rolled sheet, with an installed capacity of 800,000 tons per year [27]; industry B–that is steelwork with an installed capacity of 215 tons per hour for the production of steel, where the main processes consist of an Electric Arc Furnace for steelmaking and hot rolling [28]; industry C–that it is a lime factory, which manufactures lime by calcination of limestone in a coke kiln [29]; and industry D that is a company focused on metal waste (such as iron and aluminum) management and treatment (recovered iron is a raw material for industry B).

### 2.2. Transplantation and Sampling 

**Figure 2:**
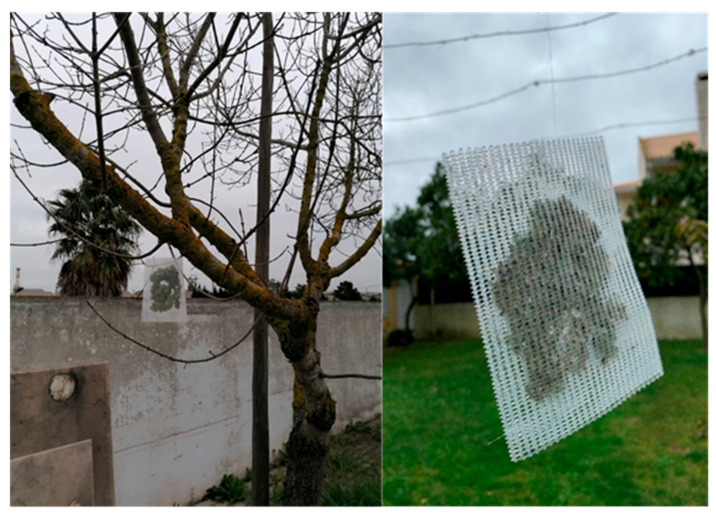
Exposed transplanted *Flavoparmelia caperata* lichens in nylon bags (**right**) that were placed in the study area (**left**).

Samples of the lichen *Flavoparmelia caperata* (L.) Hale were collected from olive trees at about 1.5 m above the soil, in Montargil (39°03′24″ N, 8°10′36″ W) (as shown in Figure 1), on 22 January 2020. Montargil is a rural area considered clean from an air pollution point of view [31]. Lichens were collected using powderless gloves and stored temporarily in paper envelopes to be transported to the laboratory. In the laboratory, four lichen samples were separated randomly, as reference base levels. Lichens were placed in nylon mesh bags and exposed in the study area, fixed to an appropriate substrate using nylon string, at about 1.8 m above the soil (Figure 2). The exposure period of the samples was approximately four and a half months (total of 137 days), from 1 February to 17 June 2020.

**Figure 3:**
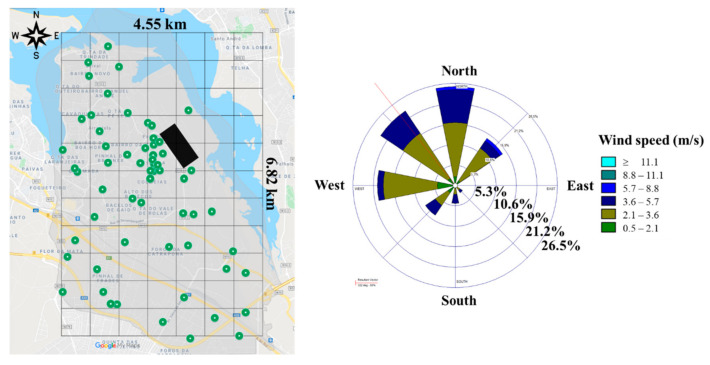
Grid of the spatial distribution of the transplanted and retrieved lichens after the exposure period (green dots) in the study area (**left**), with identification of the industrial area described before (black rectangle) (geographical data obtained from Google Earth Pro [30]), and (**right**) wind rose of the prevailing winds monitored in the weather station described in Section 2.3 (data obtained from the Portuguese Institute of Sea and Atmosphere, IPMA), during the exposure period.

A georeferenced grid of 4.55 km × 6.82 km, with 77 cells of 650 m × 620 m, was drawn for the lichens exposure, between the coordinates −9.11, 38.65 and −9.05, 38.59 (upper left and lower right corner of the grid), corresponding to the extremes of UFSAAPP (Figure 3). 

Lichens samples were distributed over the study area based on two strategies: (1) participation of the local population by exposing the transplanted lichens in outdoor of their dwellings, and (2) to have good coverage of the study area, lichen samples were exposed in selected sites to cover the established grid cells that were not covered by the strategy 1. For the population engagement, a public presentation session was held on 1 February 2020, to explain the study and to identify volunteers. A total of 88 lichen samples were exposed but only 63 could be retrieved at the end of the exposure period and subsequently analyzed (retrieved lichen samples are shown in Figure 3, left).

### 2.3. Meteorological Data

Meteorological data for the exposure period were obtained from the Barreiro-Lavradio weather station next to the study area (38°40′28″ N, 9°2′51″ W, it is the nearest weather station, and it is located at 5.5 km from the study area) and it was supplied by the Portuguese Institute of Sea and Atmosphere (Instituto Português do Mar e da Atmosfera-IPMA). 

During the exposure period, the average temperature in the study area was 16.9 ± 3.2 °C (ranging from 9.7 to 25.1 °C) and the average relative humidity was 80 ± 8% (ranging from 63 to 98%), with a total of 36 rainy days registered.

The wind rose for the exposure period is presented in Figure 3, where a predominance of winds from North (N), Northwest (NW), and West (W) was registered.

### 2.4. Chemical Analysis

At the laboratory, lichens were cleaned and cleared of extraneous material (such as dust, bark remaining, leaf debris, fungus contamination, and degraded material), and rinsed for 5 s with demineralized water, freeze-dried, and ground into powder in a ball mill with PTFE capsules under liquid nitrogen, for homogenization [32]. Pellets with an average thickness of 12 mm, were then prepared for elemental characterization by micro-X-ray Fluorescence [33]. For each lichen sample, a total of three pellet replicates were done. Figure S1 provides an overview of the procedure for the preparation of pellets.

Micro-X-ray Fluorescence (micro-XRF) analysis was conducted in the Laboratory for Heritage Science (Debrecen, Hungary), ATOMKI, Debrecen, Hungary using a Bruker M4 Tornado micro-XRF equipment (Bruker Corporation, Billerica, MA, USA). All measurements were made in a vacuum (~20 mbar). Rh excitation source set to 50 kV with 300 µA current was applied. The beam was focused down to 25 µm using polycapillary optics. To set the optimal measurement conditions, different filter combinations were applied on selected samples: (1) no filter, (2) 12.5 µm Al, (3) 100 µm Al + 25 µm Ti [34]. Test measurements to detect elements like Cd, Sn, Sb were carried out with W (50 kV, 700 µA) excitation source. Since Cd, Sn, Sb were under detection limit even with the W tube, an Rh excitation source was applied for the measurements. To achieve the result for the widest possible elemental range (Na-U), measurements were performed without a filter. 

X-ray spectra were collected simultaneously with 2 detectors (XFlash^®^ SDD with 30 mm^2^ active surfaces, Be window). Spectra and maps of ~6 mm × 7 mm areas were acquired to get the best estimate of the average composition of the samples and to eliminate the possible fluctuations in concentrations due to sampling inhomogeneity. For each lichen sample, only one of the three replicate pellets was measured. Regarding the quality control of the measurements, randomly selected pellets were re-measured 2 more times on different days with the same settings. In the case of 5 random pellets, both sides were measured, and in the case of 6 samples, all of their replicate prepared pellets were analyzed (three replicates per sample). In all cases, the resulting concentration data were within 2 sigmas. 

For the quantification, the fundamental parameter (FA) method [35] was applied using the MQuant built-in software [36]. The composition of cellulose (C_6_H_10_O_5_)_n_ was set as an “unknown” matrix. 

The elemental composition of exposed and unexposed biomonitors was assessed for a total of 20 elements (Al, As, Br, Ca, Co, Cr, Cu, Fe, K, Mg, Mn, Pb, Rb, S, Se, Si, Sr, Ti, Zn, and Zr).

### 2.5. Statistical Analysis

Statistical analysis was performed using STATISTICA software version 13. The variables in the data set exhibited a non-normal distribution, and so the Mann-Whitney U nonparametric statistics was applied [37], at a significance level of 0.05, for independent groups, to suggest whether samples come from the same population or not. One Sample Wilcoxon nonparametric test was also applied, at a significance level of 0.05, to indicate if there is a significant difference between the median of a sample group and an hypothesized value [37]. Spearman correlations [37] were used to understand the associations between parameters.

The enrichment factor (EF) is used for identifying the crustal and non-crustal origin of elements [38], and it is applied to particulate matter collected in filters [14,39], plants/lichens [32,40], and sediments [41]. The enrichment factor of the element was determined for each sample, using the reference values of soil composition (Table S1) [42] and taking Si as the crustal reference element [43], according to Equation (1):

(1) $$EF_{X}=\frac{{\left(\frac{\left[X\right]}{\left[Si\right]}\right)}_{Lichen}\;}{{\left(\frac{\left[X\right]}{\left[Si\right]}\right)}_{Soil}}$$

where ${\left(\frac{\left[X\right]}{\left[Si\right]}\right)}_{Lichen}\;$ is the ratio between the concentrations of element *X* and *Si* in the lichen and ${\left(\frac{\left[X\right]}{\left[Si\right]}\right)}_{Soil}$ is the ratio between the reference concentrations of the element *X* and *Si* in *Soil*. To account for the local variation in the soil composition in the EF calculations, elements with EF values between 1 and 10 are considered to be of a crustal origin [32], while elements with EF values greater than 10 were considered to be enriched by other sources rather than the crust [44]. 

To compare the mean elemental concentrations assessed in the exposed lichens with the unexposed lichens, the calculation of the Contamination Factor (CF) was conducted, where CF for a specific element is the ratio between the exposed and the unexposed lichen [45]. CF is also known as Exposure to Control ratio, which has been divided in five classes [46], namely (i) 0–0.25: severe loss, (ii) 0.25–0.75: loss, (iii) 0.75–1.25: normal, (iv) 1.25–1.75: accumulation, and (v) >1.75: severe accumulation.

Geostatistical modeling maps were built using ArcGIS 10.1 software (ESRI, 2020, Redlands, CA, USA), for studying the geospatial distribution of the elemental concentrations in the study area. The mapping was performed using the IDW (Inverse Distance Weighted) tool, which has been applied in similar studies [45]. IDW calculates the cell value for the unmeasured location, averaging the sampled data around each processing cell; the closer a measured point is to the center of the prediction cell, the more weight it will have [47]. In the overall equation for IDW, *v*_0_ represents the estimated value at point 0 (Equation (2)), *v_i_* the value in the known point I, *d_i_* is the distance to point I, *S* is the number of known points applied in the estimate, and *k* is assumed to equal to 2. A set of 12 neighboring samples was chosen to represent the spatial variation of the elements under study.

(2) $$v_{0}=\frac{\sum_{i=1}^{S}v_{i}\left(\frac{1}{d_{i}^{k}}\right)}{\sum_{i=1}^{S}\left(\frac{1}{d_{i}^{k}}\right)}$$

The range between the maximum and minimum concentrations obtained for each element was subdivided into five bands, corresponding to 0–20, 20–40, 40–60, 60–80, and 80–100 percentiles.

## 3. Results and Discussion

### 3.1. Elemental Characterisation

Table 1 presents the mean elemental concentrations in unexposed lichens and in the transplanted lichens after the exposure period. The major elements assessed in the exposed lichens were (by decreasing order): Ca (22.6%) > Fe (1.18%) > K (0.79%) > Si (0.62%) > Al (0.26%) > S (0.16%) > Mg (0.12%) > Ti (0.12%), with the remaining elements contributing with less than 0.10% to the total mass of the lichen samples (namely, Zn > Sr > Mn > Br > Cu > Pb > Zr > Se > Rb > Co > Cr > As). 

Compared with studies reported in the literature that used the same lichen species in industrial environments, it is possible to conclude that the concentrations found in our study area were always higher than in Sines (Portugal), an industrial area, with heavy chemical industry, a coal power plant and a deep water harbor [48,49].

It is important to highlight that the exposure period of the transplanted lichens in the present study was between February and June 2020, a period when the COVID-19 pandemic situation reached Europe. In fact, due to the global COVID-19 pandemic situation, on 19 March 2020, Portugal decreed the state of national emergency, which included mandatory confinement, movement restriction of citizens, and closure of non-essential business. As a consequence of the nationwide lockdown that occurred from 19 March to 31 May 2020, concentrations in mainland Portugal presented a mean reduction of 41% for NO_2_ and 18% for PM_10_, considering the 20 air quality monitoring stations analyzed [50], following a tendency observed all over the world [51].

In the present study area, a drastic decrease of concentrations of PM_10_ and NO_2_ was registered in April 2020 (−40.3% and −44.0%, respectively), compared to the previous six years (a significant difference, with *p*-value = 0.000) [52]. In May 2020, a lower but still significant decrease of PM_10_ (−16.7%, *p*-value = 0.000) and NO_2_ (−17.4%, *p*-value = 0.048) was also registered. These reductions were accentuated again in June 2020 for both pollutants: PM_10_ (−38.0%, *p*-value = 0.000) and NO_2_ (−29.0%, *p*-value = 0.000). Despite the reduction of emissions from anthropogenic activities (such as traffic) due to the national lockdown (which reflects in pollutants, such as PM_10_ and NO_2_), the industries (namely, steelworks) that exist in the study area never stopped operating during this period. Therefore, the present biomonitoring study has the potential to assess and evaluate the contribution of these specific industries since they were the main sources of air pollution in the area during the exposure period of the transplanted lichens.

### 3.2. Enrichment and Contamination Factors 

**Figure 4:**
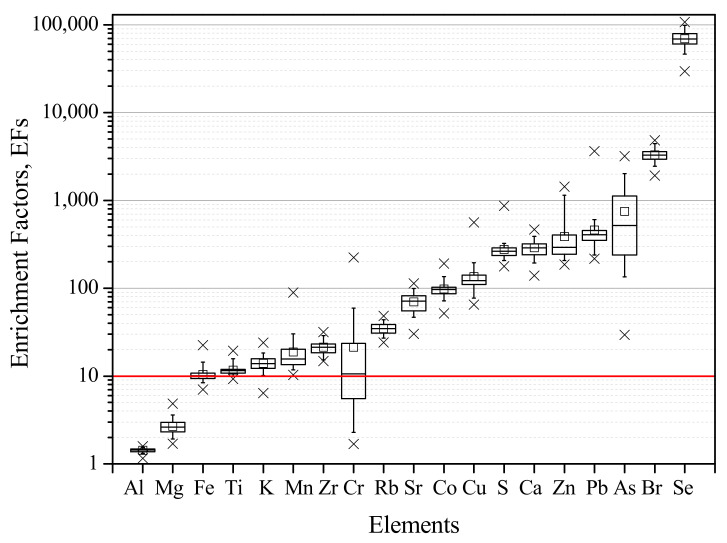
Mean EFs (and standard deviation) of the elements assessed in the exposed lichens. The red line represents the threshold of 10, the accepted minimum for the enrichment from a non-crustal source. Regarding the box plot, the square represents the mean, upper and lower times sign (×) shows the maximum and minimum values, the box provides the 25 percentile, the median and the 75% percentile, and the lower and upper minus sign (−) shows the 5 and 95 percentiles, respectively.

Figure 4 presents the mean EFs assessed for each element in the exposed lichens.

The elements Al and Mg were found to have a predominantly crustal origin (with EFs always below 10), while the remaining elements showed the contribution of non-crustal emissions to their levels. The elements Fe, Ti, and K, typically associated with a soil source [43], presented EFs slightly above 10 (with means of 10.5 ± 2.3, 11.7 ± 1.6, and 14.0 ± 2.9, respectively), indicating also anthropogenic contributions to their levels. A mix of different anthropogenic sources may have contributed to lichens enrichments, given the urban-industrial location of the study area.

**Figure 5:**
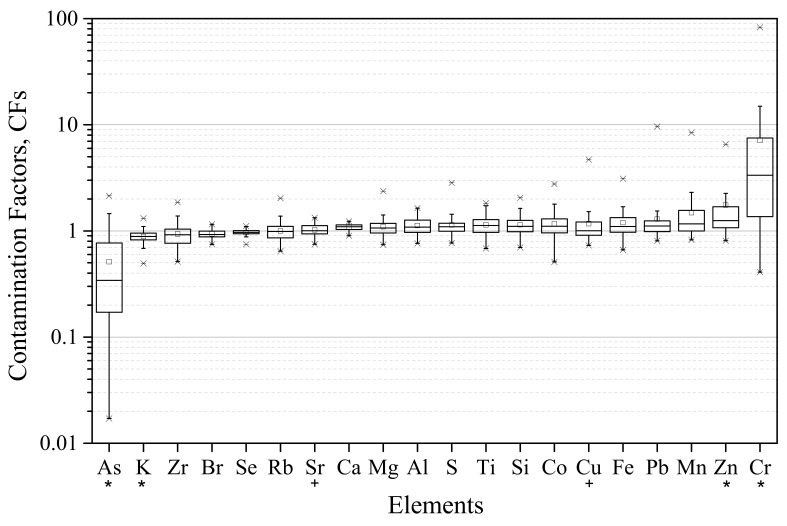
Average CFs of the elements present in the exposed samples. *–Significant statistical differences between exposed and unexposed elemental contents of lichens by a Mann-Whitney U test, at 0.05 significance level. +—Not significant statistical difference between exposed and unexposed elemental contents of lichens by the One Sample Wilcoxon test, at 0.05 significance level. Regarding the box plot, the square represents the mean, upper and lower times sign (×) shows the maximum and minimum values, the box provides the 25 percentile, the median and the 75% percentile, and the lower and upper minus sign (−) shows the 5 and 95 percentiles, respectively.

Figure 5 provides the contamination factors regarding all the studied elements and it is observed a severe accumulation for Cr (CF = 7.11 ± 0.25) and an accumulation for Zn (CF = 1.75 ± 0.43), Mn (CF = 1.48 ± 0.50) and Pb (CF = 1.29 ± 0.67). However, a significant difference between exposed and unexposed elemental contents of lichens was only found for Cr (*p*-value = 0.034) and Zn (*p*-value = 0.022), but not for Mn (*p*-value = 0.064) neither Pb (*p*-value = 0.190). However, a significant difference between the medians of elemental contents of exposed and unexposed lichens was found for all the elements, except for Cu and Sr, as ascertained by the One Sample Wilcoxon test (at 0.05 significance level). Arsenic showed a loss (CF = 0.51 ± 2.02), with significant difference between exposed and unexposed levels (*p*-value = 0.031), as well as K (CF = 0.89 ± 0.18, *p*-value = 0.033). There is evidence that metals (both mineral and PTEs) may be lost due to leaching under certain meteorological conditions [48].

The CF ratios found in the present study are within the same intervals of loss/accumulation when compared to CF ratios for lichens exposed in the urban outdoor environment [31], except for Cr and Zn, which presented higher CF ratios in the present study.

### 3.3. Identification of Emission Sources

#### 3.3.1. Spearman Correlations

Table 2 presents the positive significant Spearman correlations between the elements assessed in the exposed lichens, where values in bold represent the strongest correlations. The possible emission sources which contribute to the atmospheric PM in the study area can be qualitatively identified from the correlation matrix.

Given the significant positive correlations between Al-Si (r = 0.97), Al-Fe (r = 0.87), Al-Ti (r = 0.84), Si-Fe (r = 0.87), Si-Ti (r = 0.84), Ti-Fe (r = 0.84), it is concluded that these elements come from a same source, namely soil, since Al, Si, and Ti are typical soil elements [53,54]. In fact, Al, Fe, Si, and trace elements, such as Rb, are associated with feldspars, quartz, micas and Ti is associated with the titanite silicate [43].

In South European regions, such as Portugal, it is known that atmospheric PM can have a contribution to dust transport episodes from the interior of the Iberian Peninsula and the Saharan desert [55]. It was already found that Fe could reach high concentrations during these episodes of long-transportation of dust [56]. In fact, during the exposure period of the lichens, some Saharan dust events were identified (e.g., from 18 to 21 March 2020) [50]. 

An important amount of Al, Si, Mg, and Ti was also found in sinter plant emissions (fugitive and emissions coming from the cooling area) [1], consisting of internally mixed aluminosilicates/metallic particles, suggesting also an association between these elements and sinter plant emissions [12,53]. However, the steelworks installed in the study area do not include sintering in their processes, since scrap is used as raw material and not iron ore, and, therefore, this specific source is excluded in the study area.

Strong correlations were found between Fe-Mn (r = 0.77), Fe-Cr (r = 0.81), Cr-Mn (r = 0.92), Cr-Zn (r = 0.73), and Mn-Zn (r = 0.81) in the exposed lichens, which may indicate the existence of a common emission source, assigned to the steelworks in the study area, considering that previous studies also found strong correlations between Fe-Mn-Zn near iron and steel industry [8,57]. Typically, Fe, Cr, and Mn are considered tracers of the iron and steel industry [43]. Therefore, in the present study, despite a natural origin (soil), Fe also has the contribution of this industrial activity. This influence of different sources on Fe was found in other studies [58].

In fact, especially when scrap is employed as a raw material in the steel industry, levels of Cr and Mn can be higher [1]. Zn is also considered a tracer of the steel industry [43,59], being especially related to the use of galvanized scrap [60].

Finally, the association of Pb with the steel industry has been reported by several authors [1,61]. In the present study, significant but weak correlations between Pb-Fe (r = 0.38), Pb-Cr (r = 0.37), Pb-Mn (r = 0.41), and Pb-Zn (r = 0.48) were found, which may suggest some association of Pb emissions with the steelworks, but also the existence of other sources.

A high correlation was found for Fe and Co (r = 0.86), which suggests that Co probably is originated from the steelworks, as already found in previous studies [8].

The Electric Arc Furnace (EAF) steelmaking plants, as the one installed in the study area, typically present fugitive emissions from raw materials and slag handling and storage piles, in addition to the emission from the furnace and rolling [8].

According to the Best Available Techniques (BAT) for Iron and Steel Production [62], emissions from EAF steelmaking plants include the following metals, after abatement: Hg, Pb, Cr, Ni, Zn, Cd, and Cu (where Cr and Ni are, naturally, higher in the production of stainless steel). Typical emissions have concentrations of 0.5–50 mg dust/Nm^3^. Emissions from the secondary metallurgy (ladle metallurgy, ingot and continuous casting, oxygen blow unit) include the following metals, after abatement: Pb, Co, Ni, Se, Te, Sb, Cr, Cu, Mn, V, Sn [62].

The primary constituents of the slag produced in the EAF process are Ca, Fe, Al, Mg, Mn, Si, and Cr, where calcium and magnesium oxides are added as fluxing agents [63]. In the present study, no correlation was found between Ca-Fe, Ca-Al, Ca-Mg, Ca-Mn, Ca-Si, or Ca-Cr, and, therefore, EAF slag is not identified as a probable source.

A moderate correlation has been found between Cu-Cr (r = 0.54) and Cu-Zn (r = 0.50), which may suggest multiple emission sources. These elements are usually associated with emissions from road traffic, namely through tire (Cr, Zn) and brake wear (Zn, Cu, Ba) and motor oil (Zn) [38,43,64]. Moderate correlations between Cu, Cd, and Zn were also found regarding car traffic [65].

**Figure 6:**
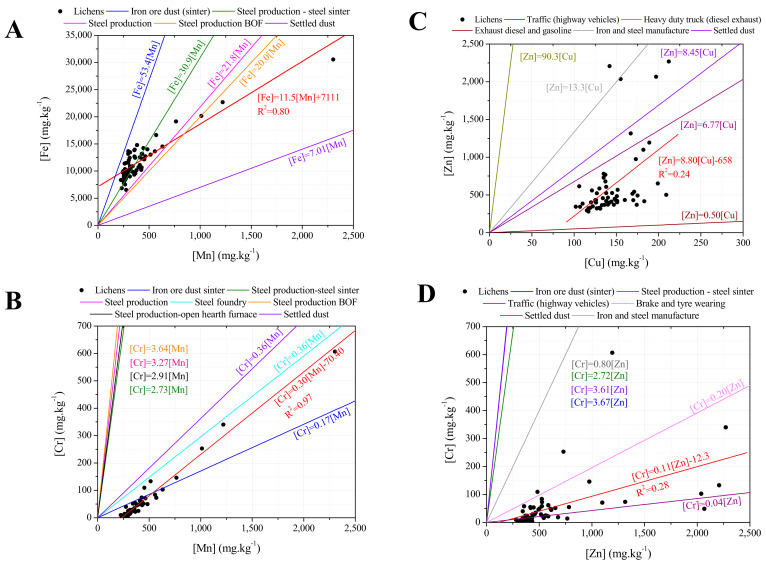
Relation between elements in the exposed lichens, in the settled dust and according to USEPA-SPECIATE and SPECIEUROPE profiles for iron and steel industry and traffic: (**A**) Mn vs. Fe, (**B**) Mn vs. Cr, (**C**) Cu vs. Zn, and (**D**) Zn vs. Cr. The red line presents the linear regression regarding the data of the exposed lichens.

USEPA-SPECIATE and SPECIEUROPE databases were used to identify emission sources, selecting source profiles from the steel industry (general and specific processes) and traffic (break and tire wearing, highway vehicles) [54]. Additionally, a comparison was also done with the elemental ratios determined in the settled dust collected in the study area in 2019 [7]. Figure 6A shows a visible high correlation between Fe and Mn concentrations assessed in the exposed lichens (r^2^ = 0.80) and a notable association to the steelmaking processes according to the databases of source profiles. It is also verified some similarity between the Fe/Mn ratio in the exposed lichens and the settled dust collected previously in the study area (11.5 and 7.0, respectively).

Figure 6B shows a very high correlation between Cr and Mn measured in lichens (r_2_ = 0.97) and a notable similarity between the Cr/Mn ratio in the exposed lichens and the settled dust (0.30 and 0.36, respectively), supporting that both elements are associated with the steelworks emissions.

Furthermore, Figure 6C,D evidence the association of Cr, Zn, and Cu to traffic emissions. Concurrently it is verified that Cr/Zn ratios in the exposed lichens and in the settled dust (0.1 and 3.6, respectively) are substantially different, suggesting that Zn emissions in the study area are substantially affected by traffic and not only by the steelworks.

#### 3.3.2. Concentrations versus Distance to Steelworks

**Figure 7:**
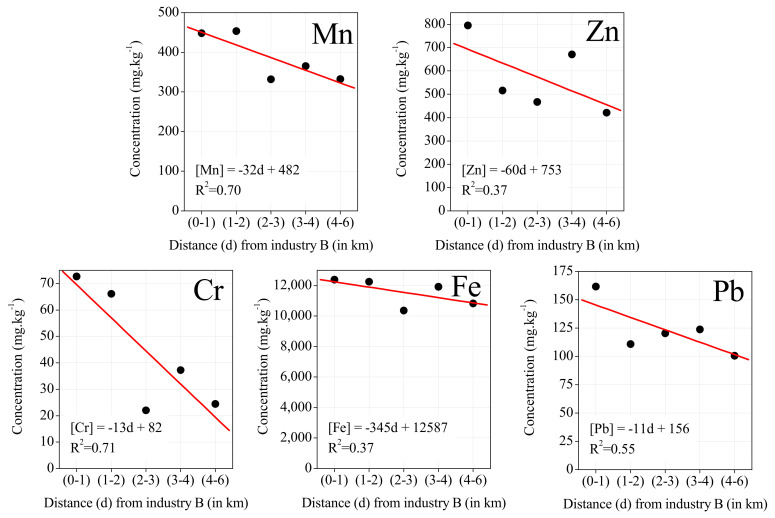
Mean elemental concentration versus distance to the steelworks.

The mean elemental concentrations were calculated for the lichens exposed in a circle with center in industry B, which is the biggest steelwork, and with 1 km radius (“(0–1)”), and in consecutive concentric crowns of 1 or 2 km radius, namely, “(1–2)”, “(2–3)”, “(3–4)” and “(4–6)”, where the edges of each interval are the distance (in km) from the established center in industry B. Figure 7 shows the mean concentrations of selected elements regarding the distance from industry B. It should be mentioned that there is a distance of around 2 km from both steelworks in the study area (namely, industries A and B), and thus both facilities are included (and are expected to directly influence the samples) in the 2 km radius (namely the samples located in “(0–1)” and “(1–2)”).

As expected, elements associated with the steel industry (Fe, Cr, Mn, Zn, and Pb) registered the highest concentrations in the lichens exposed near the steelworks, within a 1 or 2 km radius from industry B, and with concentrations decreasing with distance, as shown by Figure 7. Mean Zn levels showed a peak at 4 km from industry B, and this may reflect the traffic influence since it is in the distance range of the nearby highway.

Comparing the mean concentrations of exposed lichens in the 1 km radius circle, to the control sample exposed at a 6580 m distance from industry B, levels decreased more sharply for Cr (6.1 times), than for Zn (2.5), Pb (1.9), Mn (1.7), and Fe (1.5).

**Figure 8:**
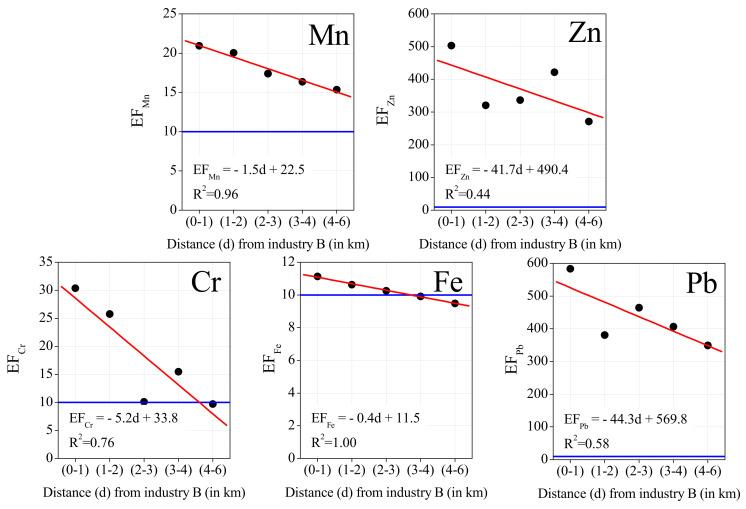
Mean Enrichment Factors (EFs) versus distance to the steelworks. The blue line represents the threshold of 10, the accepted minimum for the enrichment from a non-crustal source.

The same analysis regarding the variability of EFs with the distance to industry B was also conducted, as shown by Figure 8. The same trend was observed, namely, the EFs of Mn and Fe decrease on a regular basis with the distance; in the case of Zn and Cr, there is an increase in the EF at a distance of 3–4 km from the industry B, consistent with the traffic highway influence.

**Figure 9:**
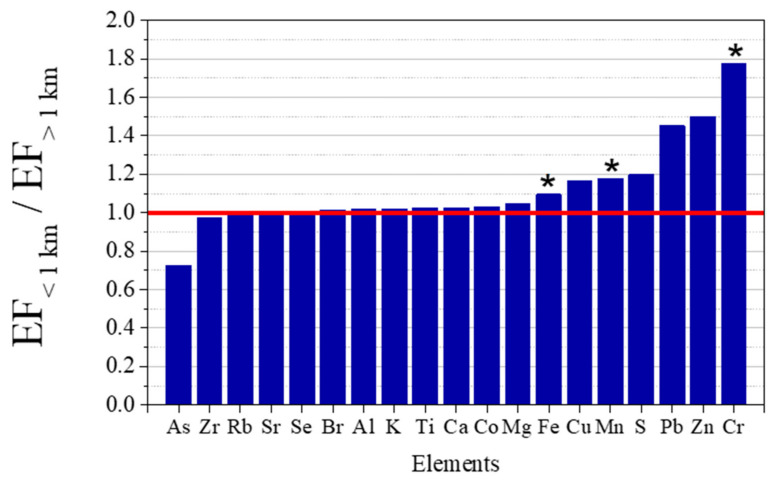
Ratio between EF in samples located at less than and more than 1 km from steelworks. * Significant statistical difference between the EF in the two groups by a Mann-Whitney U test, at 0.05 significance level.

The ratio between the EFs for samples exposed at a distance less than and more than 1 km from the steelworks’ area is shown in Figure 9. This ratio is higher for Cr (1.77), Zn (1.50), and Pb (1.45), i.e., close to steelworks there was an increase in EF for these elements, as well as for Mn and Fe. Nevertheless, only the EFs of the elements Cr (*p* = 0.015), Mn (*p* = 0.035), and Fe (*p* = 0.031) presented significant differences, between the samples located at less than and more than 1 km from the steelworks.

#### 3.3.3. Spatial Distribution Patterns

**Figure 10:**
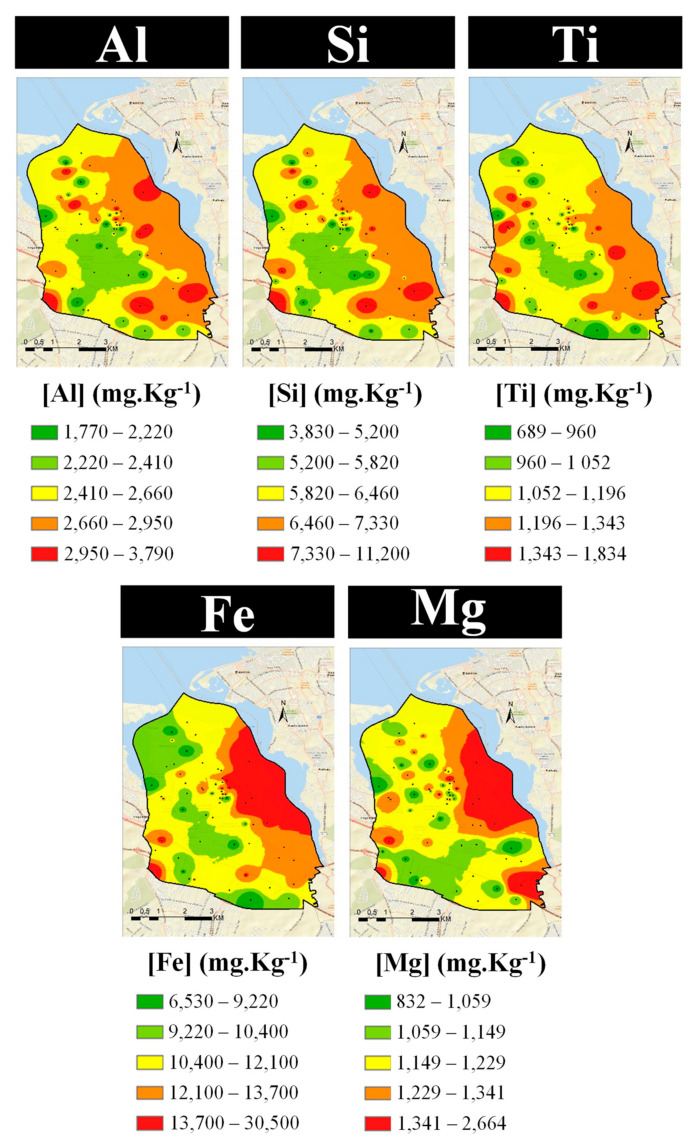
Spatial distribution of soil-related elements in the study area: (**top**) natural origin and (**bottom**) a mixture of natural and anthropogenic origins.

**Figure 11:**
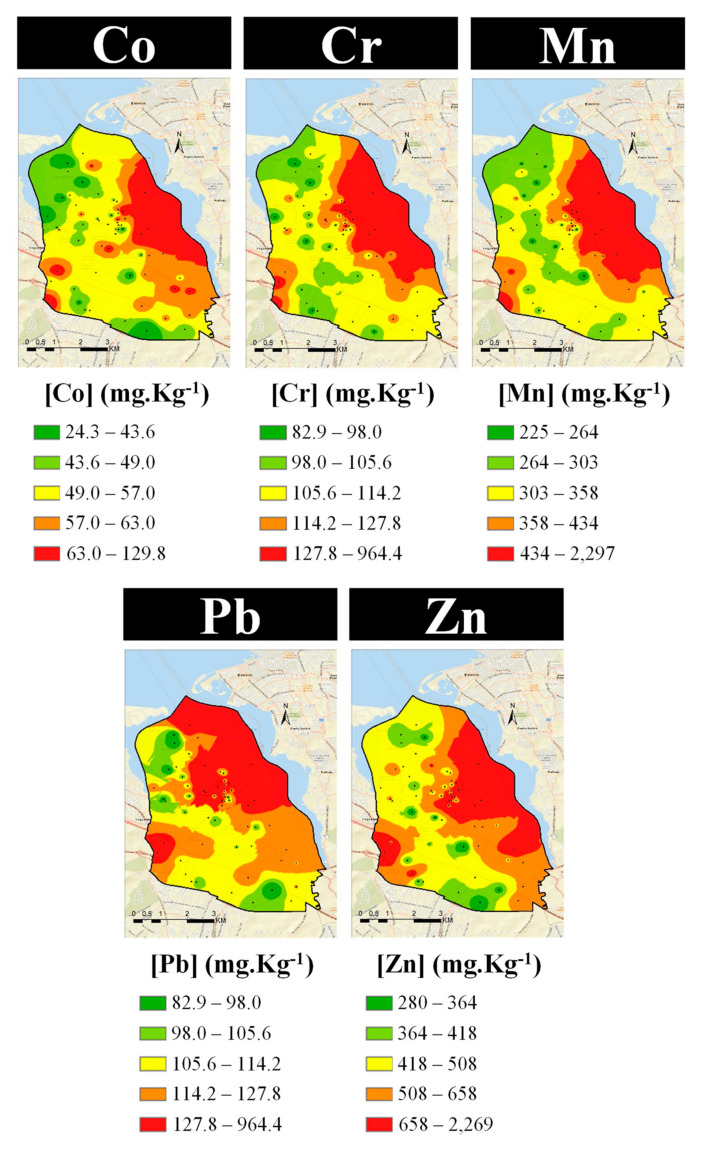
Spatial distribution of elements related with anthropogenic origin, namely industry.

**Figure 12:**
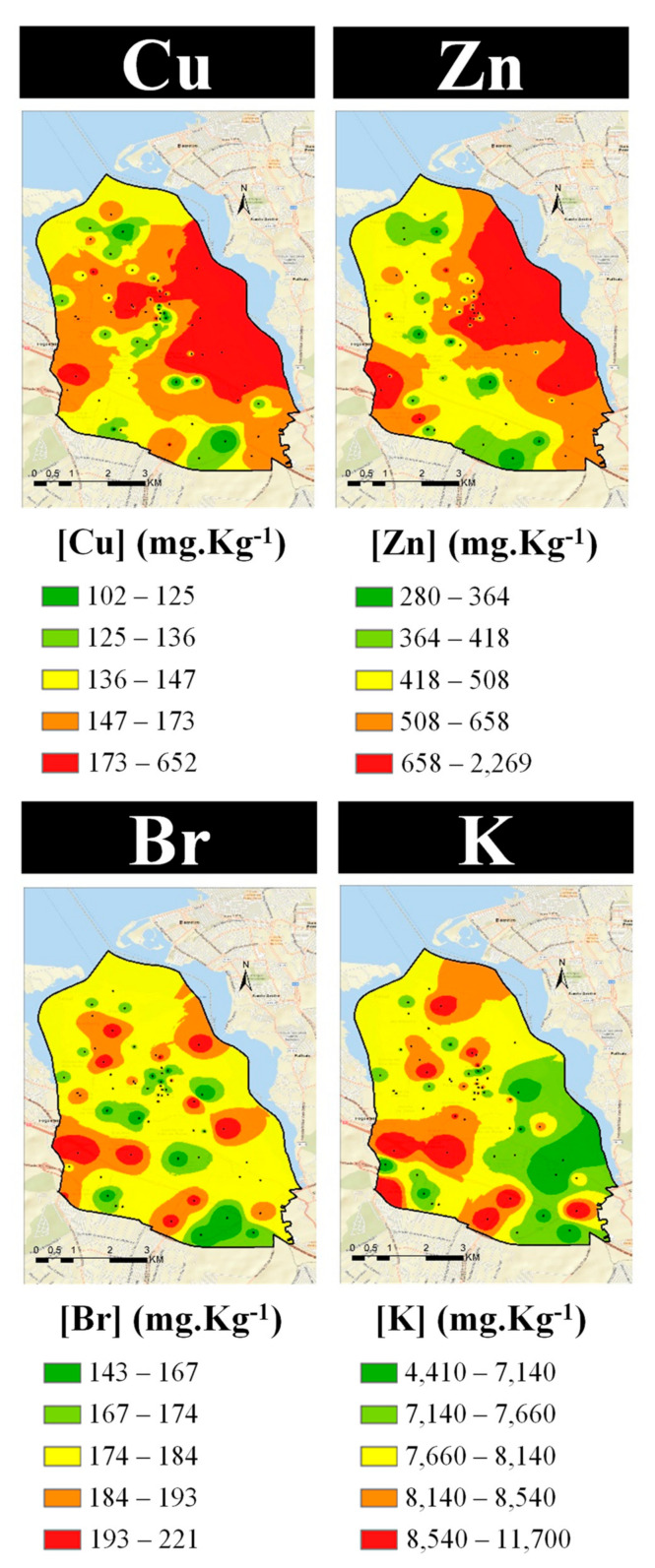
Spatial distribution of elements related to an anthropogenic origin, namely from road traffic (**top**) and biomass burning (**bottom**).

As a final step in the identification of emission sources, the spatial distribution of elemental concentrations in the exposed lichens was mapped (Figure 10, Figure 11 and Figure 12). The similarities of the spatial distributions obtained between elements and the correlations found above, support the grouping of elements by types of emission source: natural origin from the soil (Al, Si, and Ti—Figure 10, top); a mixture of soil with natural and anthropogenic origins (Fe and Mg—Figure 10, bottom); anthropogenic resulting from the industrial activity, namely steelworks (Co, Cr, Mn, Pb, and Zn—Figure 11); anthropogenic from road traffic (Cu and Zn—Figure 12, top) and biomass burning (Br and K—Figure 12, bottom).

There is a close resemblance of the spatial distributions of soil origin elements Al, Si, and Ti, as expected from the high correlation coefficients obtained for these elements. The relatively high concentrations of these elements nearby the steelworks is certainly due to the resuspension of particles of geological origin, by the wind and heavy duty traffic [66].

In the studied area, Fe and Mg concentrations may result from the natural emission of soil and the activity of the steelworks, which may provide these elements from fugitive emissions. This hypothesis is supported by the similarity of the spatial distribution of these elements compared to Co, Cr, Mn, Cr, and Zn, associated with the steelworks. Regarding Pb, an additional hot spot can be observed in the north of the study area, but no industry could be identified. Additionally, the spatial distribution of Cu evidences high concentrations near roads due to the influence of traffic [43].

As the wind blew predominantly from the north (N), northwest (NW), and west (W) (Figure 3) during the exposure period, the highest concentrations of the elements related to the steelworks would be expected downwind of this emission source, that is in the south-southeast sector of the sampling grid, as is indeed apparent from the spatial distribution in Figure 11.

The S enrichment in the industrial area may be associated with metal smelting [43] and, in fact, good correlations were obtained above for S-Fe (r = 0.62), S-Cr (r = 0.62), and S-Mn (r = 0.62); but may also derive from local emissions of SOx related to coal/coke burning [43], since there is a coke kiln at a lime company located in the steelworks park-industry C. The significant correlation S-Cu (r = 0.52) also suggests some association with traffic emissions [12], which may be due to heavy-duty traffic in the steelworks area (Figure 12).

Elements Br and K present a spatial distribution with some similarities (and a significant correlation, r = 0.54), indicating a common anthropogenic source, such as biomass burning [43]. This is in accordance with the analysis of the national inventory of emissions for the municipality of Seixal, which identified residential combustion as one of the main emission sources in the region, concerning both PM_10_ and PM_2.5_, besides industry and road transport [67].

Figure S2 provides the spatial distribution in the study area of the chemical elements that were not associated with an identified source, such as Ca. Lime and calcium carbide are mineral additions used in the production process of industry B [28], and industry C is a factory of lime products and derivatives, with both industries located in the steelworks park. Nevertheless, high levels of Ca were not obtained in the steelworks area, but in the southeast of the grid (Figure S2). Since there is no cement industry or quarry in the study area, Ca enrichment is probably due to the physiological characteristics of the lichens [32], as granules with calcium oxalate may occur in lichens due to environmental factors [68].

The spatial distribution of the elements discussed previously is in accordance with the Air Quality Map of Seixal, according to which the highest values of particulate matter (PM_10_ and PM_2.5_) were registered in the industrial areas and main roads of the municipality [67]. The authors concluded that the air quality index regarding PM_10_ is “average” generically throughout the municipality, except in the industrial area where it is “weak” or “bad”; for PM_2.5_, the air quality index varies from generically “weak” to “very bad” in the industrial area.

## 4. Conclusions

The present study aimed to perform a biomonitoring study of air pollution using lichens in an urban-industrial area where the local population has expressed several concerns regarding air quality in recent years. The methodology applied in this study allowed to involve the local population and to obtain a spatial distribution of the chemical elements absorbed by the lichens during a period where the activities in the area were mainly the industries, due to the COVID-19 national lockdown.

Using a set of different analysis approaches to the chemical content of the exposed transplanted lichens in the study area, it was possible to identify hotspots of air pollution in the area (taking advantage of the possibility to assess the spatial variability due to the use of a grid of biomonitors) and to define the potential sources affecting the local air quality. 

A total of five different sources were identified as contributing to the local air quality: (i) a soil source of natural origin (based on Al, Si, and Ti), (ii) a soil source of natural and anthropogenic origins (based on Fe and Mg), (iii) a source from the local industrial activity, namely steelworks (based on Co, Cr, Mn, Pb, and Zn); (iv) a source from the road traffic (based on Cr, Cu, and Zn), and (v) a source of biomass burning (based on Br and K).

The impact of the industries located in the study area on the local air quality was identified (namely, the steelworks). To identify the quantitative contribution of such sources and to design targeted mitigation measures, future efforts should be conducted to assess particulate matter levels by reference methods, which will allow a more comprehensive and quantitative analysis of the issue, taking into account the national and European legislation.

Despite biomonitoring being a technique known for decades, this study showed again the potentialities and advantages of such an approach (used as a complementary strategy to reference methodologies), providing spatial information of air pollution. This information is crucial to define future strategies, based on knowledge: understand exposure levels of the population to air pollution, their potential health impacts, and to define mitigation measures to improve air quality.

## Figures and Tables

**Table 1:** Mean elemental mass fractions (in mg·Kg^−1^, dry weight) in unexposed and exposed lichens samples and comparison with values from other studies.

	Present Study						(Godinho et al., 2009) [48]	(Pacheco et al., 2008) [49]
	Unexposed Lichens			Exposed Lichens			Industrial Area, Sines ^1^	Industrial Area, Sines ^1^
Element	Mean ± SD	Min	Max	Mean ± SD	Min	Max	Mean ± SD	Mean ± SD
Al	2310 ± 190	2140	2550	2590 ± 450	1770	3790	1040 ± 146	3170
As	58.5 ± 24.8	36.0	88.0	30.0 ± 28.4	1	125	0.33 ± 0.11	1.33
Br	192 ± 17	170	211	180 ± 16	143	221	14.6 ± 1.8	13.7
Ca	208,000 ± 23,000	180,000	232,000	226,000 ± 15,000	187,000	257,000	8890 ± 1690	4390
Co	47.0 ± 10.5	32.0	56.0	54.9 ± 16.2	24	130	0.68 ± 0.16	1.33
Cr	7.33 ± 0.58	7.00	8.00	52.1 ± 90.8	3	607	6.4 ± 0.4	7.1
Cu	140 ± 16	122	158	163 ± 90	102	654	n.d.	n.d.
Fe	9860 ± 1480	8430	11,700	11,800 ± 3800	6500	30,600	651 ± 137	3190
K	8910 ± 690	8200	9710	7900 ± 1120	4410	11,750	3877 ± 737	4170
Mg	1130 ± 90	1020	1220	1230 ± 270	830	2670	992 ± 79	1320
Mn	274 ± 33	246	314	406 ± 297	225	2303	24.0 ± 0.5	28.2
Pb	101 ± 15	82	117	130 ± 111	81	967	n.d.	n.d.
Rb	69.8 ± 17.0	48.0	86.0	69.7 ± 15.1	45	142	4.4 ± 1.3	23.4
S	1390 ± 120	1280	1560	1580 ± 410	1070	3950	n.d.	n.d.
Se	77.5 ± 6.0	58.0	86.0	74.7 ± 5.7	58	86	n.d.	0.49
Si	5460 ± 370	5010	5790	6240 ± 1280	3810	11,220	n.d.	n.d.
Sr	553 ± 36	508	586	565 ± 74	415	737	n.d.	n.d.
Ti	1010 ± 100	910	1140	1150 ± 230	690	1840	102 ± 1	274
Zn	347 ± 16	333	365	608 ± 454	280	2270	140 ± 34	134
Zr	83.8 ± 17.1	59.0	98.0	78.5 ± 19.7	43	156	n.d.	n.d.

^1^ after 4-month exposure; SD—standard deviation; n.d.—not determined.

**Table 2:** Spearman correlations between elements in the exposed lichens, significant at the 0.05 level. Values in bold represent strong correlations (correlation coefficient above 0.70), while weak and medium correlations refer to correlation coefficients between 0.30–0.49 and 0.50–0.70, respectively.

Elements	Al	As	Br	Ca	Co	Cr	Cu	Fe	K	Mg	Mn	Pb	Rb	S	Se	Si	Ti	Zn	Zr
Al			0.33		**0.71**	0.62		**0.87**	0.29	0.50	0.56		0.66	0.56		**0.97**	**0.84**	0.42	0.67
As																			0.52
Br							0.43	0.32	0.54	0.42	0.25		0.46	0.49	0.35	0.39	0.37		0.38
Ca																			
Co						0.64	0.27	**0.86**	0.28	0.41	0.59	0.34	0.62	0.49		**0.72**	0.64	0.44	0.60
Cr							0.54	**0.81**		0.39	**0.92**	0.37	0.32	0.62		0.62	0.64	**0.73**	0.42
Cu								0.39		0.35	0.57	0.32		0.52		0.32	0.37	0.50	0.34
Fe										0.51	**0.77**	0.38	0.67	0.62		**0.87**	**0.84**	0.60	0.65
K										0.44			0.61	0.36		0.35	0.33		0.34
Mg											0.44		0.46	0.48		0.48	0.50	0.29	0.57
Mn												0.41	0.33	0.62		0.58	0.62	**0.81**	0.43
Pb														0.27				0.48	
Rb														0.48		0.66	**0.73**	0.27	0.58
S																0.61	0.61	0.58	0.41
Si																	**0.84**	0.42	0.68
Ti																		0.55	0.66
Zn																			0.29

## Data Availability

The datasets generated and analysed during the current study are not publicly available but are available from the corresponding author on reasonable request.

## Supplementary Materials

The following supporting information can be downloaded at: https://www.mdpi.com/article/10.3390/ijerph19031364/s1, Figure S1: Procedure for preparation of pellets: (**A**) used ball mill RETSCH, (**B**) homogeneised powder of lichen sample, (**C**) used Pelletiser SPECAC, and (**D**) pellet of a lichen sample. Figure S2. Spatial distribution of elements without a specific identified source. Table S1. Reference values of soil composition (in mg·Kg^−1^) defined by Mason and Moore [42], used for the calculation of the Enrichment factors.
