# Supplementary material for: Spatial Distribution of Air Pollution, Hotspots and Sources in an Urban-Industrial Area in the Lisbon Metropolitan Area, Portugal—A Biomonitoring Approach
Source: Int J Environ Res Public Health. 2022 Jan 26;19(3):1364. doi: 10.3390/ijerph19031364 (PMC8834712; doi:10.3390/ijerph19031364)
Supplement: Supplementary file 1 [file ijerph-19-01364-s001.zip › ijerph-1499815-supplementary.pdf]

5. Supplementary Information

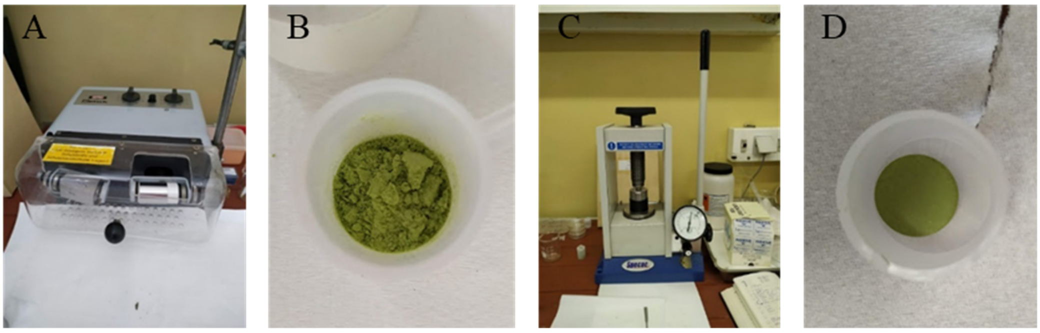

Figure S1. Procedure for preparation of pellets: (A) used ball mill RETSCH, (B) homogeneised powder of lichen sample, (C) used Pelletiser SPECAC, and (D) pellet of a lichen sample.

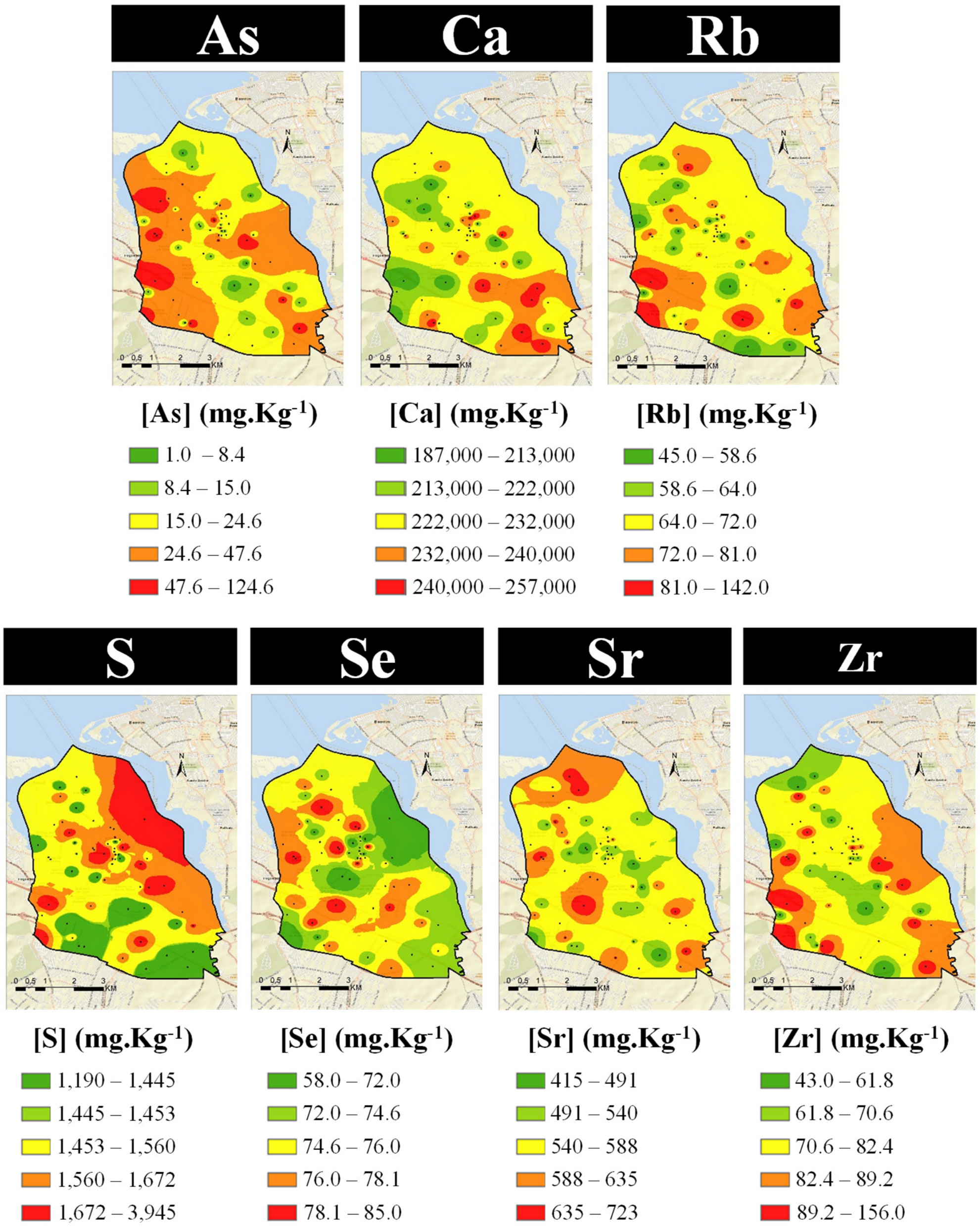

Figure S2. Spatial distribution of elements without a specific identified source.

Table S1. Reference values of soil composition (in $mg.Kg^{-1}$) defined by Mason and Moore [42], used for the calculation of the Enrichment factors.

Element	Mean crustal concentration ($mg.Kg^{-1}$)
Al	81300
As	1.8
Br	2.5
Ca	36300
Co	25
Cr	100
Cu	55
Fe	50000
K	25900
Mg	20900
Mn	950
Pb	13
Rb	90
S	260
Se	0.05
Si	277200
Sr	375
Ti	4400
Zn	70
Zr	165
